# Intra-Gene DNA Methylation Variability Is a Clinically Independent Prognostic Marker in Women’s Cancers

**DOI:** 10.1371/journal.pone.0143178

**Published:** 2015-12-02

**Authors:** Thomas E. Bartlett, Allison Jones, Ellen L. Goode, Brooke L. Fridley, Julie M. Cunningham, Els M. J. J. Berns, Elisabeth Wik, Helga B. Salvesen, Ben Davidson, Claes G. Trope, Sandrina Lambrechts, Ignace Vergote, Martin Widschwendter

**Affiliations:** 1 Department of Women’s Cancer, Elizabeth Garrett Anderson Institute for Women’s Health, University College London, London, United Kingdom; 2 Deparment of Mathematics, University College London, London, United Kingdom; 3 CoMPLEX, University College London, London, United Kingdom; 4 Department of Health Sciences Research, Mayo Clinic College of Medicine, Rochester, MN, United States of America; 5 Department of Biostatistics, University of Kansas Medical Center, Kansas City, KS, United States of America; 6 Department of Laboratory Medicine and Pathology, Mayo Clinic, Rochester, MN, United States of America; 7 Department of Medical Oncology, Erasmus MC-Cancer Center, Rotterdam, The Netherlands; 8 Department of Obstetrics and Gynaecology, Haukeland University Hospital, Bergen, Norway; 9 Department of Pathology, Oslo University Hospital, Norwegian Radium Hospital, University of Oslo, Faculty of Medicine, Institute of Clinical Medicine, Oslo, Norway; 10 Department of Gynaecological Oncology, Oslo University Hospital, Norwegian Radium Hospital, Oslo, Norway; 11 Division of Gynecologic Oncology, Department of Obstetrics and Gynecology and Leuven Cancer Institute, University Hospitals Leuven, Katholieke Universiteit Leuven, Leuven, Belgium; Peking University Cancer Hospital and Institute, CHINA

## Abstract

We introduce a novel per-gene measure of intra-gene DNA methylation variability (IGV) based on the Illumina Infinium HumanMethylation450 platform, which is prognostic independently of well-known predictors of clinical outcome. Using IGV, we derive a robust gene-panel prognostic signature for ovarian cancer (OC, *n* = 221), which validates in two independent data sets from Mayo Clinic (*n* = 198) and TCGA (*n* = 358), with significance of *p* = 0.004 in both sets. The OC prognostic signature gene-panel is comprised of four gene groups, which represent distinct biological processes. We show the IGV measurements of these gene groups are most likely a reflection of a mixture of intra-tumour heterogeneity and transcription factor (TF) binding/activity. IGV can be used to predict clinical outcome in patients individually, providing a surrogate read-out of hard-to-measure disease processes.

## Introduction

Differences in DNA methylation (DNAm) levels are amongst the earliest changes in human carcinogenesis [1] and are a hallmark of cancer [2], offering the potential for novel strategies to predict cancer biology and outcome. The epigenetic differences which these changes give rise to are more stable than differences in gene expression level. Gene expression levels, as measured by RNA, are subject to periodic and transient variability (such as diurnal variation and mRNA instability), which do not apply to DNAm. Identifying reliable indicators of differences in DNAm patterns might provide a valuable lead for the development of DNA-based cancer biomarkers in tissue and bodily fluids.

Ovarian cancer (OC) and endometrial cancer (EC) are the most common gynaecological cancers [3]. Only one in three patients with advanced stage OC survive for five years after their initial diagnosis [4]. Very little is known about OC biology and how to manipulate this disease therapeutically. DNAm changes are important in cancer [5]; the epigenome is an interface between the genome and the environment [6, 7], and hence DNAm changes can measure exposure to environmental risk factors of cancer. DNAm biomarkers which represent a surrogate for patterns of gene interaction have previously been associated with clinical outcome in a wide variety of cancers [8], as well as specifically in women’s cancers [9].

**Fig 1:**
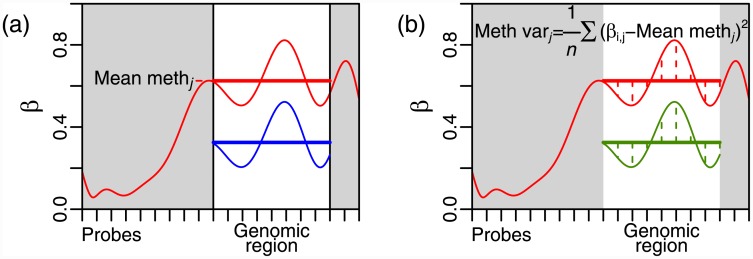
Per-gene methylation measures. (a) The mean methylation level over a specific genomic region is calculated separately for the TSS200 (promoter) and gene body genomic regions. The blue curve indicates the new position of the red curve after an additive global shift in methylation level, which might be due to technological or other experimental factors, and the difference between the horizontal red and blue lines (mean levels) illustrates the effect of this shift on the mean methylation level. (b) The intra-gene methylation variability (IGV) is calculated from the variation around the mean methylation level, i.e., from the dashed vertical lines, and is similarly calculated separately for the TSS200 and gene body genomic regions. The vertical green lines are changed very little compared to the vertical red lines, illustrating that such a global additive shift in mean methylation level has much less effect on IGV, which is therefore referred to as a ‘self-calibrating measure’.

Sample to sample variability of DNAm at specific genomic locations is known to be important in the development of cancer [10, 11], and it has recently been shown that an increase in intra-gene variability of DNAm (IGV), a measure of within-sample methylation variability (Fig 1a), is highly associated with cancerous tissues in comparison to healthy [12]. Differential methylation is the commonly-used method by which methylation levels are compared between tissues, phenotypes and experimental conditions (equivalently to differential expression of genes). Here, we develop a prognostic signature based on IGV which is independent of well-known clinical prognostic features, and show that this IGV prognostic signature is likely a surrogate readout reflecting a mixture of intra-tumour heterogeneity and transcription factor (TF) binding/activity.

## Results

### Comparison of predictive robustness of per-gene methylation measures in data

To assess the effectiveness and robustness of IGV compared to mean methylation levels, we compared four per-gene methylation measures, based on mean methylation level and IGV (Fig 1). For each gene, we calculated mean methylation level and IGV, separately for the promoter (TSS200) and gene body regions, by using the Illumina Infinium HumanMethylation450 platform specifications of the CpGs in these regions for each gene. We considered different genomic regions separately, because methylation patterns vary greatly from one genomic region to another, and the effect of methylation level on gene regulation varies according to genomic region. The four measures we compared, are as follows:

- TSS200 mean methylation
- TSS200 IGV
- Gene body mean methylation
- Gene body IGV

We obtained genome-wide DNAm profiles, via the Illumina Infinium HumanMethylation450 platform, from 218 primary OC samples. For each of the four measures described, we used ‘Elastic Net’ [13, 14] to find a prognostic selection of genes. Elastic net has been found to be an optimal linear modelling method to identify groups of genes which act together as part of a common biological process [15]. It is a regression method which ‘chooses’ the set of genes which model the data best, trying to include as few genes in the model as possible, whilst ensuring that the model predicts the outcome of interest as accurately as possible. In doing so, it discards genes which do not provide useful information, or which provide repeated information. As our aim is to find a minimal set of genes to use as a prognostic signature, it is important to note that amongst these genes, there will be groups of genes for which their IGV contains redundant or overlapping information, and there will be groups of genes for which IGV contains complementary information for each gene. Hence we chose to use the Elastic Net technique to accurately discern such a non-redundant grouping of genes as a minimal predictive set from very many possibilities, genome wide. We note that whilst this methodology may seem complex in this context, simpler methodology would not be able to discern these parsimonious groupings of genes in which overlapping and redundant information is kept to a minimum.

**Fig 2:**
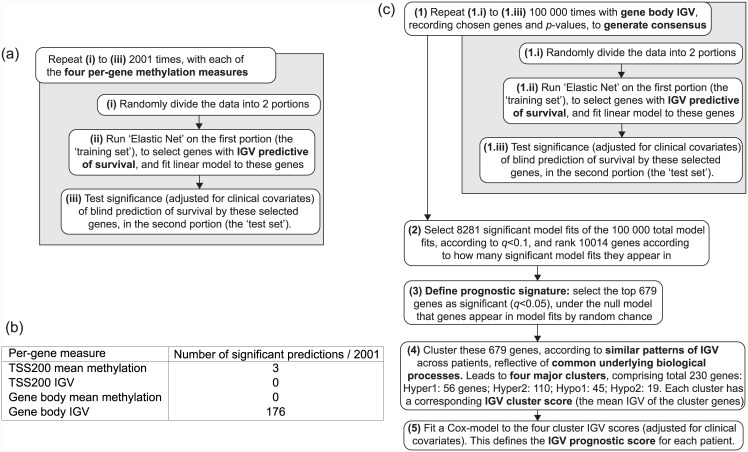
Overview of methods. (a) Methodology overview for comparison of the four per-gene methylation measures. (b) Results of this comparison. (c) Methodology overview for calculation of ovarian cancer IGV prognostic score.

We assessed the effectiveness of the per-gene methylation measures as prognostic measures by randomly dividing the data into two portions: a ‘training set’, and a ‘test set’. Elastic Net was used to select genes and fit a model to the training set, and the ability of this gene selection and model to blindly predict patient survival outcome (adjusted for clinical covariates) was assessed using the test-set. This was repeated 2001 times, and significantly predictive selected groups of genes were defined according to false discovery rate (FDR) adjusted [16] *p*-value (i.e., FDR *q*-value) < 0.1 (Fig 2a). As shown in Fig 2b, only gene body IGV predicts well.

### Derivation of an ovarian cancer prognostic signature, and IGV prognostic score

We used IGV to derive an OC DNAm prognostic signature (Fig 2c), based on gene-body IGV (from here on simply referred to as ‘IGV’). We did this by determining a consensus on a set of genes predictive of survival, by following the same procedure of splitting data into test and training sets, and then assessing the gene selection and fitted model for their ability to blindly predict patient survival outcome (adjusted for clinical covariates) in the test set. In order to ensure convergence to a stable result, we made 10^5^ such partitions of the data, each resulting in a predictive selection of genes. Of these, 8281 were found as significant (FDR *q* < 0.1), and significance for each gene was then calculated based on the number of significant models in which that gene appeared. 679 genes were selected like this for inclusion in the OC prognostic signature at a significance level of FDR *q* < 0.05, with the least significant gene present in 1057 out of 8281 model fits. The top 100 most significant of these genes are shown in Supplementary Tables (S1 File).

Genes often act together as part of biological pathways, and processes. Hence, we can expect that these 679 OC prognostic signature genes can be represented by a smaller number of underlying biological processes, which are important to disease progression. Grouping genes with similar experimental measurements by using clustering methodology is well established as an effective approach for determining clinically relevant prognostic markers [17, 18]. Hence, to uncover such groupings in the 679 genes of our OC prognostic signature, we carried out consensus clustering [19], to identify groups of genes with similar patterns of IGV across patients. Each cluster identified in this way reveals a different IGV trend, and therefore may correspond to a different underlying biological process, which gives rise to the pattern of IGV observed in that cluster. The clustering was carried out separately for genes which were individually associated with worse patient survival outcome for increased IGV (‘hyper’ genes) and for decreased IGV (‘hypo’ genes). The result was four clusters: two from the hyper genes, called clusters ‘hyper 1’ and ‘hyper 2’, and two from the hypo genes, called clusters ‘hypo 1’ and ‘hypo 2’; they are shown in Supplementary Tables (S1 File). The mean IGV of the genes of each of the four clusters gives an IGV ‘cluster score’, for each cluster and for each patient, which are taken to be representative of the different IGV trends, and corresponding underlying biological processes, within the OC prognostic signature.

We then calculated an IGV prognostic score, by fitting a multivariate Cox proportional hazards model (accounting also for clinical covariates) to the four IGV cluster scores. It was not possible to fit such a model to the full set of 10014 genes, because there are many more predictor variables (genes) than samples [20]. However, reducing the prognostic signature to 4 cluster scores, i.e., 4 predictors, allows the Cox proportional hazards model to be fitted. This results in a model coefficient for each cluster score/predictor; these are used to calculate the IGV prognostic score. The IGV prognostic score is a one-number prognostic indicator for a single sample/patient, and we note that it must be calculated based on all four cluster scores, to be significantly prognostic.

**Fig 3:**
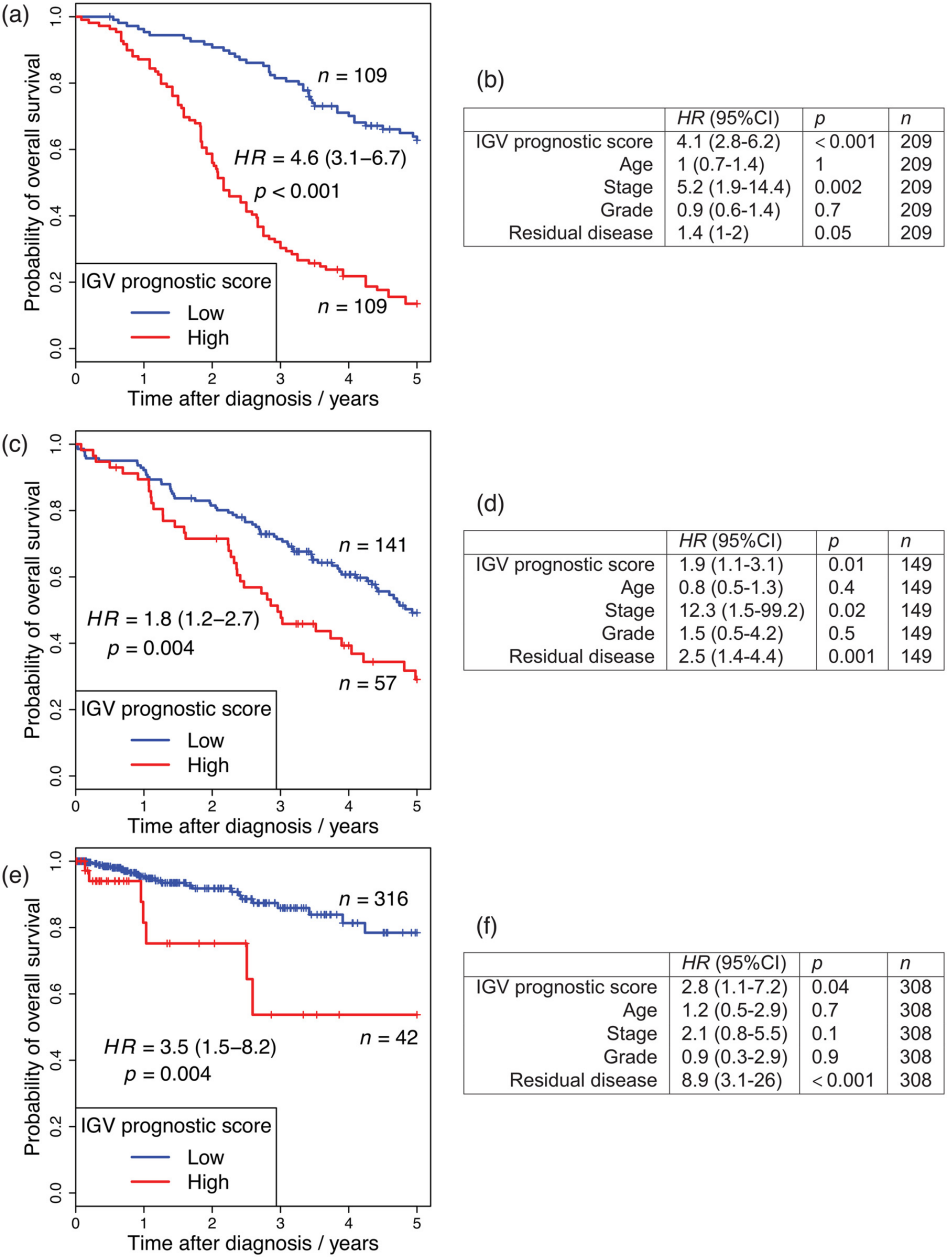
IGV OC prognostic signature validation. (a), (c) and (e): Comparison of survival curves of groups defined by the IGV prognostic score, in: (a) the main OC data set, (c) the Mayo Clinic OC validation set, (e) the uterine cancer TCGA validation set. The groups are divided by the median IGV prognostic score derived in the main OC DNAm data-set. The hazard ratio (*HR*) is displayed with 95% C.I. in brackets, with corresponding *p*-value calculated by univariate Cox regression. (d), (e) and (f): Multivariate Cox regression comparing the same groups defined by the IGV prognostic score.

The median of this IGV prognostic score was used to divide the patients of the main OC data set into better and worse prognostic groups, shown in Fig 3a and 3b. The IGV prognostic score was validated in two independent sets of cancers derived from the Mullerian tract. A new OC set from the Mayo Clinic (*n* = 198) confirmed the prognostic capacity of the IGV prognostic score in both univariate (Fig 3c) and multivariate (Fig 3d) analyses. In order to test whether the IGV prognostic score is only limited to OC, or whether it is also predictive in other cancers which arise from the same embryological structure (i.e., the Mullerian duct), we applied our prognostic score to a publically available uterine corpus endometrioid carcinoma (UCEC) set from *The Cancer Genome Atlas* (TCGA) [21] (*n* = 358). Again, in both univariate (Fig 3e) and multivariate (Fig 3f) analyses, we were able validate the IGV prognostic score.

We note that using the median prognostic score from the main OC data-set (the training set) to dichotomise the patients of the Mayo OC and TCGA UCEC validation sets makes this a true assessment of the prognostic ability of this methodology. This is because by this method, the patients of the validation sets are classified one by one into a better or worse prognostic group, in terms of their DNAm measurements only. This classification is done according to a threshold or boundary dividing these prognostic groups (i.e., the median of the prognostic score in the training data-set), and this threshold is set entirely independently of these validation data-sets.

### IGV and intra-tumour heterogeneity

**Fig 4:**
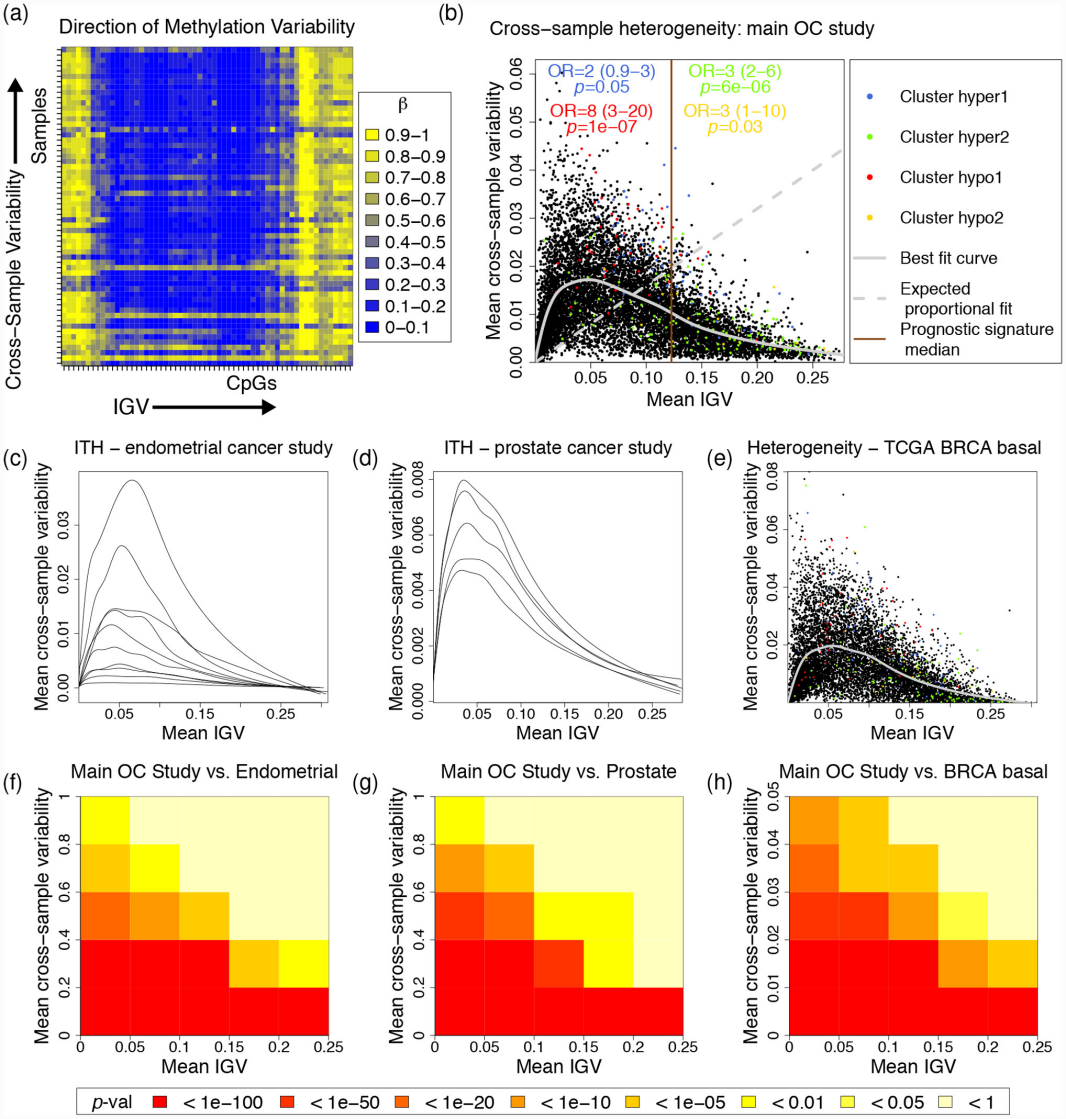
Comparison of IGV with Intra-Tumour Heterogeneity. (a) Cross-sample variability of methylation (Intra-tumour heterogeneity) and IGV are calculated in different and complementary directions. The heatmap displays the methylation profile of a single gene (horizontal axis), across multiple samples (vertical axis). (b)-(e) A characteristic pattern of high cross-sample variability (intra-tumour heterogeneity) when IGV is low, and vice-versa, is consistently observed across different studies: (b) Main OC data-set, (c) Endometrial cancer intra-tumour heterogeneity data-set, (d) prostate cancer intra-tumour heterogeneity data-set, (e) BRCA basal data-set. (f)-(h) The overlap of genes in each region of (b) with genes in equivalent regions of (c)-(e) is highly significant. In (c) and (d), each line relates to samples from a single patient, and is a best fit curve equivalent to that shown in (b) and (e). In (b), odds-ratios and *p*-values at the top of the plot show enrichment of the genes of each cluster, either side of the median IGV of the prognostic signature. Abbreviations: ITH (intra-tumour heterogeneity), OC (ovarian carcinoma), BRCA (breast cancer invasive carcinoma).

We suggest that the IGV cluster scores are each representative of different biological processes, important for disease outcome. But what are these processes? To try to find some answers to this question, we first hypothesised that intra-tumour heterogeneity might be a reflection of IGV. The subject of intra-tumour heterogeneity is currently receiving a great deal of attention, uncovering much spatial and temporal diversity in genomic processes within individual tumours [22]. Ideally, the DNA methylome of individual cells from the same tumour sample should be analysed to address this question. As an alternative approach, we use here cross-sample methylation variance (i.e., mean methylation variance of individual CpGs of a specific gene-body region), as a measure of intra-tumour methylation heterogeneity, in order to assess how this varies as a function of IGV (Fig 4a). Cross-sample methylation variability is also a measure of how similar the methylation profiles are for the gene, across samples. If cross-sample methylation variability were a reflection of IGV, as IGV increases, we would expect to see a consistently increasing cross-sample methylation variance (Fig 4b, expected proportional fit). However, instead we see a pattern in which for low IGV, cross-sample methylation variance increases, whereas for high IGV, cross-sample methylation variance decreases again and is very low for the highest IGV values. In order to validate this further, we analysed two additional data sets, for which several samples from different regions of the same cancer have been taken. The first additional data-set is derived from endometrial cancers, where independent samples have been taken from 2 or 3 primary cancer and metastatic sites, in each of 10 patients (Fig 4c, one curve of best fit is shown per patient). The second is derived from prostate cancers, where 8 independent samples have been taken from the same tumour, from each of five cancer patients [23] (Fig 4d, one curve per patient). The pattern of these curves is almost identical to the intra-tumour heterogeneity studies, in the main OC study which we used to identify the OC prognostic signature (Fig 4b), and in basal samples from the TCGA breast-cancer invasive carcinoma (BRCA) data-set (Fig 4e). The overlap of genes in all regions of these plots is also highly significant across data sets (Fig 4f–4h).

The genes of cluster hyper 1 are somewhat over-represented in the left half of Fig 4b, where IGV is lower, and cross-sample methylation heterogeneity is typically higher. This suggests that the increased IGV of these genes is associated with intra-tumour heterogeneity. However, the genes of clusters hyper 2 and hypo 2 fall mostly in the region of high IGV and low cross-sample methylation variability (towards the right of Fig 4b). This means that, for the genes of these clusters, their methylation profiles tend to be similar in different samples from the same tumour, or from different tumours. In the case of cluster hyper 2, this corresponds to high methylation variability within a single gene in poor prognostic cases, and that this variability is consistently similar throughout the tumour and between tumours. Hence, the genes of cluster hyper 2 show high IGV in poor prognostic cases, yet appear to be independent of intra-tumour heterogeneity. Therefore, we speculate that the increased IGV of these genes is a tumour-cell inherent phenomenon, independent of intra-tumour heterogeneity. This means that the IGV prognostic signature combines measures of intra-tumour heterogeneity, with those of independent, tumour-cell inherent phenomena. We note that the terms ‘hyper’ and ‘hypo’, here relate to change, rather than absolute level. For example, S1 Fig shows that cluster hypo 2 has the highest IGV of any cluster; however, the IGV of this cluster is actually lower in poor compared to good prognostic cases.

**Fig 5:**
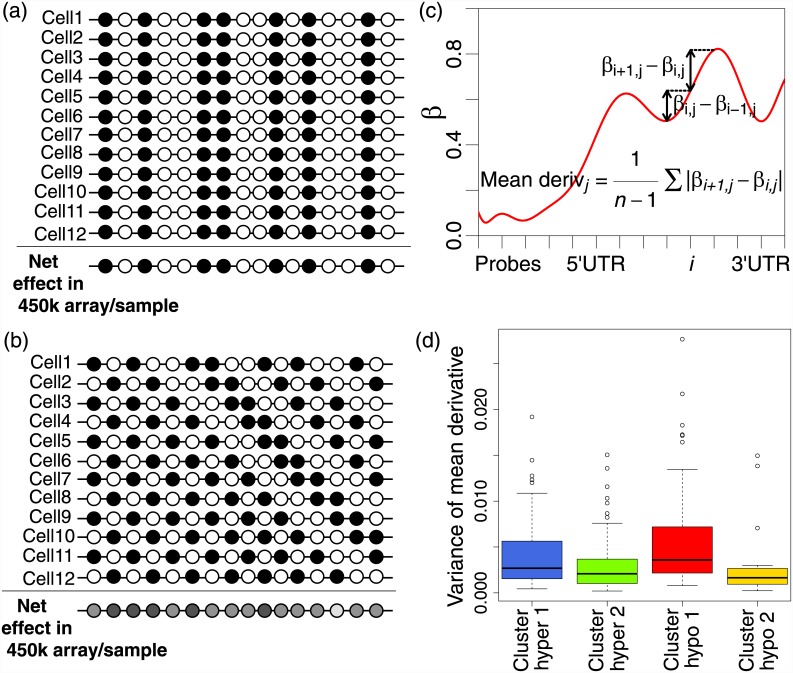
Heterogeneity and the effects of cell mixing on the 450K array. The 450K array provides methylation measurements from a mixed-up sample of multiple cells. (a) An example of a methylation pattern which is highly variable, in a similar way across cells. This leads to low cross-sample heterogeneity, and high IGV, as in cluster hyper 2. (b) An example of a methylation pattern which is highly variable, but in a heterogenous way across cells. This leads to high cross-sample heterogeneity, however the net effect of averaging the methylation profiles across the mixed up sample of many cells gives a measurement with low IGV, as in cluster hypo 1. (c) A measure of CpG-CpG methylation variability, calculated as the mean derivative, or the mean absolute difference in methylation level between adjacent CpGs. (d) The variability of the mean-derivative measure across samples quantifies the heterogeneity of the CpG-CpG methylation variability. Cluster hyper 2 is low according to (d), and hence corresponds to a pattern such as (a). Cluster hypo 1 is high according to (d), and hence corresponds to a pattern such as (b).

The genes defining cluster hypo 1 have the highest mean cross-sample methylation variability (Fig 4), as well as the highest mean methylation level (S2 Fig), and the low IGV of the hypo 1 genes is associated with poor prognosis. At first, it seems difficult to explain that poor prognostic cancers have lower IGV in the hypo1 genes, yet these hypo1 genes also represent high sample-sample methylation heterogeneity. To explain this, we used a measure of CpG-CpG methylation variability, which we call the mean derivative [12], which is calculated as the average absolute difference in methylation levels between adjacent CpGs of the gene-body of a gene, in a single sample. The Illumina HumanMethylation 450K array measures the methylation levels of specific CpG loci, averaged across a mixed-up sample of many cells. Fig 5a and 5b shows two examples of how high methylation variability at the single-cell level might manifest in measurements acquired using this technology.

In the example of Fig 5a, we see that there is little cell-cell heterogeneity, although there is much variability within a gene. Hence, this results in measurements of high IGV, and low cross-sample methylation variability, as we see in cluster hyper 2. Then Fig 5b shows an example in which there is much cell-cell variability, as well as much variability within a gene. The result is that the cross-sample methylation variability of the array measurements is high, but because the highly variable methylation profiles ‘average out’ across the mixed-up cells in the sample, the net result is a measurement with low IGV. To examine whether this hypothesis is plausible, we use the mean derivative measure of CpG-CpG methylation variability (Fig 5c). By considering how heterogenous this CpG-CpG variability is across samples (Fig 5d), we are able to confirm that in the genes of cluster hypo 1, the CpG-CpG methylation variability tends to be more different across different cells than in any other cluster, as reflected by the high variance of the mean-derivative measurements. We are also able to confirm from Fig 5d that in the genes of cluster hyper 2, the CpG-CpG methylation variability tends to be less different across different cells than in any other cluster, as indicated by the low variance of the mean derivative. Hence, these data support the model shown in Fig 5a and 5b for genes in cluster hyper 2 and hypo 1, respectively.

### Functional role of transcription-factor activity in IGV

**Fig 6:**
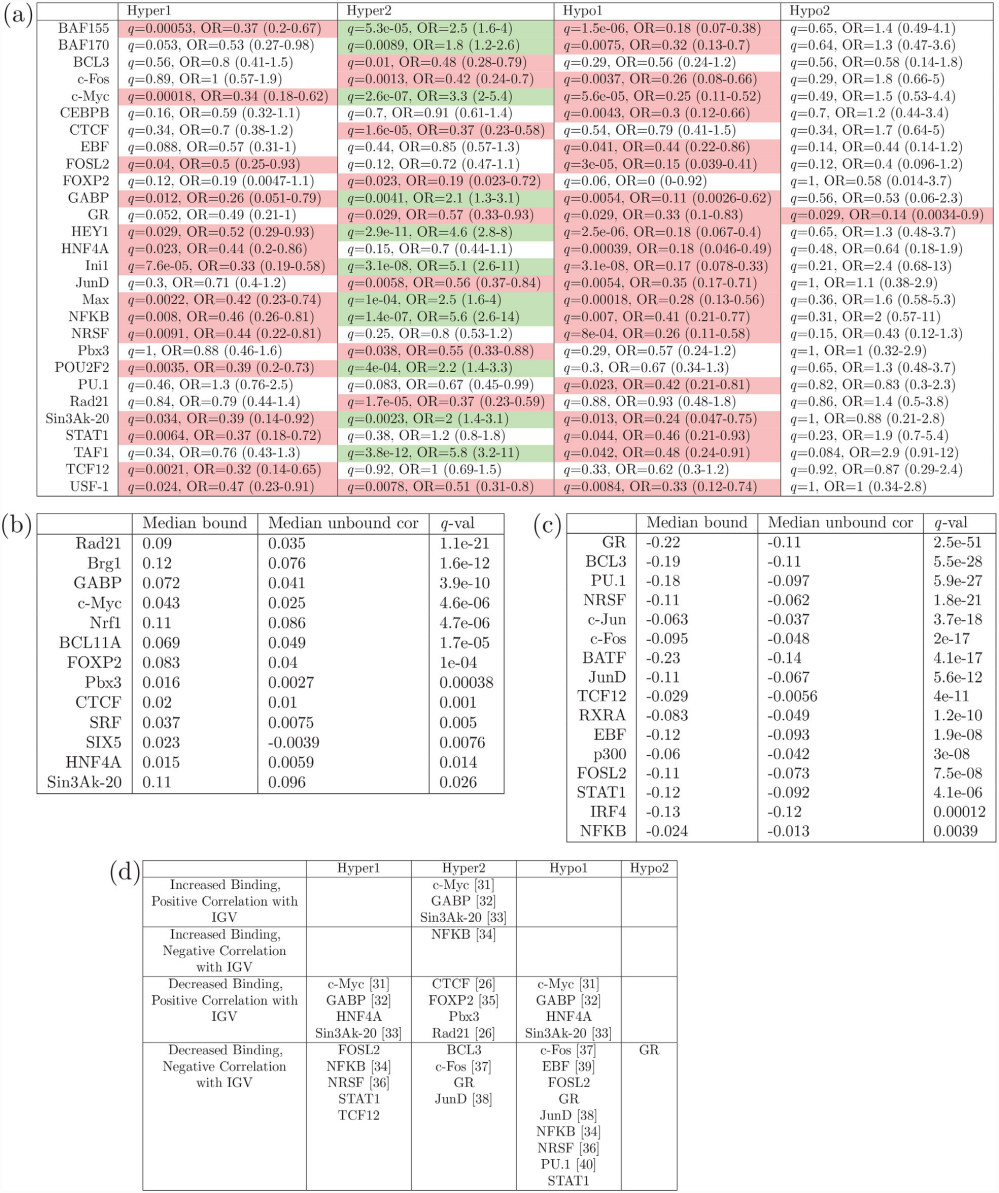
Transcription Factor Binding and Expression Correlation with IGV. (a) False discovery rate adjusted *p*-values and odds-ratios (OR) show enrichment of binding of specific transcription factors (TFs), to the gene body regions of the genes of each cluster. TFs for which binding is significantly over or under enriched (Fisher’s exact test, FDR *q* < 0.05) are coloured green and red, respectively. (b) TFs which show significantly more positive correlation with IGV of the genes they bind to, compared to the genes they do not bind to. (c) TFs which show significantly more negative correlation with IGV of the genes they bind to, compared to the genes they do not bind to. (d) TFs which are significant according to (a) and either (b) or (c); TFs with known relevance are indicated with a reference to the relevant study. The lack of enrichment of TF binding to the genes of cluster hypo2, is a reflection of the small number (19) of genes in this cluster.

As the genes comprising cluster hyper 2 seem to show the same IGV in most cells of the tumour, but the high IGV of the cluster hyper 2 genes is associated with poor prognosis, we deem the cluster hyper 2 IGV to be a ‘consistent tumour-cell inherent phenomenon’, which is likely to be regulated by differential binding of transcription factors (TF). Therefore, we examined TF binding to the gene body regions of the OC prognostic signature genes, and tested the correlation of TF expression with the IGV of the genes they bind to (in a TCGA set of basal breast cancers). We found that each prognostic signature cluster shows its own distinctive pattern of TF binding (Fig 6a), which we can hypothesise is associated with the biological processes responsible for the characteristic pattern of IGV observed in that cluster.

Transcription factor binding site information, obtained from the ENCODE (Encyclopedia of DNA Elements) project [24], was available for the gene body regions of all the genes represented on the Illumina HumanMethylation 450K array, for 55 transcription factors. We tested each of these 55 TFs, for significantly increased or decreased binding to the genes of each prognostic signature cluster. Cluster hypo 2 only consists of 19 genes, and hence we would not expect to see many significant correlations, due to small sample size. But interestingly, for cluster hyper 2 (comprised of genes whose methylation levels vary little across tumours but show higher IGV), we see that 20% (11/55) of the TFs tested show significantly more binding to these genes than expected, whereas 16% show significantly less binding than expected. For the gene clusters for which DNAm varies across/within tumours and have generally low IGV (clusters hyper 1 and hypo 1), not a single TF showed higher than expected binding, whereas 27% and 38% of TFs show lower than expected binding to the genes comprising cluster hyper 1 and hypo 1, respectively. This is consistent with the idea that TF binding is involved in distinct and different processes associated with IGV and methylation heterogeneity within a sample.

We also wanted to test the actual correlation of expression of the TFs with IGV of the genes they bind to, and genes they do not bind to, genome-wide. To do this, we used a TCGA set of basal breast cancers, for which 450k methylation data as well as expression data exist. We have already established a high degree of similarity in behaviour of our prognostic signature genes in OC and these TCGA BRCA basal samples (Fig 4). Further, it has been comprehensively demonstrated by the TCGA consortium that high-grade serous ovarian and uterine and BRCA basal cancers are extremely molecularly similar [25]. Fig 6b and 6c show TFs with significantly more positive, and more negative, correlation with IGV of the genes they bind to, compared to the genes they do not. It is interesting that the two most highly ranked transcription factors according to increased positive correlation of their expression with IGV in bound genes, *Rad21* and *Brg1* (*SMARCA4*), are both parts of chromatin modifying complexes with relevance to stem cell identity [26, 27]. In particular, *Brg1* (*SMARCA4*) has been shown recently to have particular relevance to small-cell ovarian cancer [28–30]. The overlap between the TFs which show significantly different binding patterns in relation to the OC prognostic signature genes, and TFs which display significantly altered correlation of their expression with IGV of genes they bind to, is shown in Fig 6d. Much relevant detail has already been reported about most of these TFs (references noted in the figure): either their binding is influenced by methylation (or *vice-versa*), or they are involved with chromatin remodelling in stem cells. The TFs shown in Fig 6d are important to the processes underlying disease progression, which are associated with our OC prognostic signature (TFs with known relevance are indicated with a reference to the relevant study [26, 31–40]). Therefore we hypothesise that IGV, in our OC prognostic signature gene panel, represents a surrogate measure for their activity and role in disease transformation.

### Association of prognostic signature CpGs with CpG islands and enhancer regions

The location of CpGs relative to CpG islands (CGIs) is known to be an important determinant of the functional role of these CpGs [41]. We tested for enrichment of probes annotated to the CGI regions ‘island’, ‘shore’ and ‘shelf’ amongst all gene body annotated probes, as well as probes annotated to gene bodies of the genes of our prognostic signature, and of the four clusters. While we found that gene body probes were overall significantly depleted for probes in these CGI regions, the opposite was true for gene bodies of our prognostic signature (see Supplementary Tables in S1 File). This effect appears to be largely driven by the second cluster. This indicates a prominent role for CpG islands in the relevant areas of the genes of our prognostic signature.

Location of CpGs relative to enhancer regions is also known to be relevant to the functional role of CpGs. We tested whether there was enrichment of methylation sites annotated to enhancers in gene bodies in general, finding that there is, as would be expected. Then, we tested enhancer enrichment similarly in the prognostic signature gene bodies, and the gene bodies of the individual clusters. We found that there is an even greater enrichment in the prognostic signature gene bodies than in gene bodies in general, which is consistent with IGV being mediated by transcription factor binding. This effect seems to be driven particularly by the ‘hypo’ clusters, for which methylation variability decreases with worse prognosis. These results are shown in Supplementary Tables (S1 File).

## Discussion

We have found that IGV (a per-gene measure of intra-gene variability of DNAm) is a far more robust prognostic marker tool than mean methylation levels: Fig 2b indicates that gene body IGV has the potential to become an effective prognostic tool. While it is true that the Illumina HumanMethylation 450K array provides more DNAm measurements for the gene-body than for any other genomic region, and hence gene-body derived measures can potentially provide more information than those derived from the promoter region when using this technology, this is unlikely to be the whole explanation for its effectiveness in this study.

We note that it has previously been found that the most variably methylated CpGs occur more frequently in gene bodies than in promoters [42]. However, while it is well established that promoter methylation in CpG-dense regions is associated with gene repression [41], the effects of gene-body methylation are less clear. Gene body methylation has recently been shown to have a direct effect on gene expression level [43], however it may also be associated with other influences on transcription and translation, such as prevalence of alternatively spliced gene products [41]. Findings are also starting to emerge that gene-body methylation may be an effective therapeutic target in cancer [43].

The OC prognostic signature we have developed based on IGV, is able to blindly predict patient prognostic outcome in two independent data sets from studies by the Mayo Clinic and TCGA (*n* = 198 and *n* = 358, respectively), with highly statistically significantly different clinical outcomes between these groups (*p* = 0.004 in both data sets). The methodology we present here is, after calibration on a training data-set, able to classify patients one by one without reference to any more new samples into better and worse prognostic groups. Thus, our method gives a prediction of better or worse prognosis individually to patients. For this reason, it can be considered to be a true prognostic measure.

It is becoming increasingly clear that understanding intra-tumour heterogeneity, is crucial to understanding cancer biology [22, 23], including ovarian cancer [44], and recent work has shown the effectiveness of intra-tumour heterogeneity as a prognostic marker [45]. Asking the question, what is IGV, we examined whether intra-tumour methylation heterogeneity might be a reflection of IGV, finding that while for genes with relatively low IGV this may be true, for genes with high IGV, intra-tumour methylation heterogeneity does not appear to reflect IGV. Therefore, we may hypothesise that in these genes, IGV represents a tumour-cell inherent phenomenon. Investigating further the reasons for this phenomenon, by looking at binding of TFs and the correlation of their expression with IGV of genes they bind to, revealed a distinctive pattern of TF binding to different groups of genes, and identified a panel of TFs which are highly associated with prognostic IGV. However, the TF binding maps we analysed here is not exhaustive, and so this picture can be expected to become fuller, as more such TF binding data become available. We have also found evidence of the importance of CpG islands to the functional role of IGV in the genes of our prognostic signature and clusters.

Cancer is a heterogenous disease, which can, even within the same tissue type, show very different molecular characteristics between patients. Hence, it is becoming clear that for our mechanistic understanding of cancer to progress, we must focus on large-scale data-sets (i.e, ‘big-data’), which are able to capture such heterogeneity with sufficient statistical power [45, 46]. Such analyses require computational statistical tools which are relatively new to medical science, which in turn requires interdisciplinary collaboration. In this study, we have made use of several such tools, to derive our prognostic signature gene-panel, and then to identify common molecular patterns within this gene-panel, which reflect heterogenous oncogenic processes. The methodology we present here is computationally efficient, and would naturally scale well to larger data-sets, and would be applicable to analysis of cancer data from a wide range of tissues of origin.

We have conclusively demonstrated that our OC prognostic signature is an effective and robust prognostic tool, and we also hypothesise that it is an easy to measure surrogate for disease processes mediated by specific transcription factors. IGV is a robust prognostic marker, which is independent of known clinical prognostic factors.

## Methods

### Data and preprocessing

The main ovarian cancer (OC) data set, which was used to derive the OC prognostic signature, consists of 221 samples each of which was taken from a different patient, of whom 158 died from the disease before the end of the study. For each sample, a DNA methylation profile collected via the Illumina Infinium HumanMethylation450 platform was available, together with information on the clinical variables survival status (alive or not), survival time (i.e., time to last follow up or time to death), disease stage (I-IV), disease grade (1–3), and residual disease status (present or not). 3 samples were removed due to missing clinical data, leaving the the *n* = 218 samples used to derive the OC prognostic signature. A further 9 samples were excluded from the multivariate analysis of the IGV prognostic score, due to additional missing clinical data.

An independent data set from a study of OC carried out by the Mayo Clinic was used for validation of the OC prognostic signature. Data from this study similarly included a DNA methylation profile for each sample collected via the Illumina Infinium HumanMethylation450 platform; clinical data was also available for this data set for the same variables as the main OC data set. There were *n* = 198 samples in this data set, of whom 115 died from the disease before the end of the study. 49 samples were excluded from the multivariate analysis of the IGV prognostic score, due to missing clinical data.

An additional independent data set from a study of uterine corpus endometrioid carcinoma (UCEC) for further validation of the OC prognostic signature was downloaded with the *The Cancer Genome Atlas* (TCGA) project [21]. Data from this study similarly included a DNA methylation profile for each sample collected via the Illumina Infinium HumanMethylation450 platform, which was downloaded at level 3; clinical data was also downloaded if possible for each sample for the same variables as the OC data set. There were 358 samples in this data set, of whom 32 died from the disease before the end of the study. 50 samples were excluded from the multivariate analysis of the IGV prognostic score, due to missing clinical data.

For the intra-tumour heterogeneity analysis, we considered two data sets, one from endometrial cancer (EC) (samples from multiple metastatic sites for each of 10 patients), and one from prostate cancer [23] (multiple samples from the same tumour for each of 5 patients). For comparison with cross-patient methylation heterogeneity, we downloaded DNAm data for breast cancer invasive carcinoma (BRCA) basal samples from TCGA (42 samples). Each of these data-sets included a DNA methylation profile for each sample collected via the Illumina Infinium HumanMethylation450 platform, again downloaded at level 3 for the TCGA BRCA data-set. For the gene expression analysis in BRCA basal samples, we downloaded gene expression data for the same 42 samples from TCGA, at level 3.

Probes with non-unique mappings and which map to SNPs had already been removed from the UCEC and BRCA TCGA DNAm data before they were downloaded, and these same probes were also removed from the other DNAm data sets. Probes mapping to sex chromosomes were also removed (by prior agreement); in total 98384 probes were removed from the DNAm data sets, of the 482421 probes originally present on the array. After removal of these probes, 270985 probes with known gene annotations remained. Individually for each data set, probes were then removed if they had less than 95% coverage across samples; probe values were also replaced if they had corresponding detection *p*-value greater than 5%, by KNN (*k* nearest neighbour) imputation (*k* = 5).

A summary of the data-sets analysed here appears in Table 1. A detailed summaries of the patient cohorts of the main ovarian and uterine cancer DNA methylation data-sets analysed here appear in Table 2.

**Table 1:** Data-sets analysed.

Data-set	Patients	Samples per patient	Deaths	Removed
Main OC DNAm	221	NA	158	12
Mayo OC DNAm	198	NA	115	49
TCGA UCEC DNAm	358	NA	32	50
Endometrial ITH DNAm	10	2–3	NA	NA
Prostate ITH DNAm	5	16	NA	NA
TCGA BRCA basal DNAm	42	NA	NA	NA
TCGA BRCA basal Expr	42	NA	NA	NA

Abbreviations: ITH, intra-tumour heterogeneity; DNAm, DNA methylation; OC, ovarian cancer; UCEC, uterine corpus endometrial carcinoma; BRCA, breast cancer invasive carcinoma.

**Table 2:** Patient cohort details of the main DNA methylation data-sets analysed.

Data-set	Total patients	Stage 3–4	Grade 3	Age 60 or over	Residual disease
Main OC	221	183 (83%)	144 (65%)	94 (43%)	92 (42%)
Mayo OC	198	158 (80%)	164 (83%)	114 (58%)	70 (35%)
TCGA UCEC	358	103 (28%)	226 (63%)	241 (67%)	47 (18%)

### Per-gene methylation measures

Four per-gene measures were tested, as follows:

- **TSS200 mean** The mean methylation level of the probes annotated to the TSS200 region, which is the region within 200bp upstream of the TSS (transcriptional start site); approximately the promoter region.
- **TSS200 IGV** The variance of the methylation level of the probes annotated to the TSS200 region.
- **Gene body mean** The mean methylation level of the probes annotated to the gene body.
- **Gene body IGV** The variance of the methylation level of the probes annotated to the gene body.

To calculate these measures, annotation information specifying which probes map to each gene and genomic region was used, as downloaded from Gene Expression Omnibus (GEO) [47], and as part of the *R* / *Bioconductor* software package *IlluminaHumanMethylation450k*. The mean methylation was calculated for genes with any number of probes annotated to the relevant genomic region (12970 and 15839 genes for TSS200 and gene body respectively). The methylation variance was calculated for genes with at least 3 probes annotated to the relevant genomic region (7557 and 10014 genes for TSS200 and gene body respectively).

### Cross-validation to compare per-gene methylation measures and derive OC prognostic signature

The samples (patients) of the main OC data-set were randomly split in to a ‘training set’ (2/3 of the data, 145 samples) and a ‘test set’ (the remaining 1/3 of the data, 73 samples). The Elastic Net [13, 14] was used to select a prognostic group of genes and fit a predictive model to these genes based on the training set; this model was then assessed using the test set. This was repeated 2001 times for each of the four per-gene methylation measures.

As the aim here is to predict clinical outcome, the Elastic Net was used in its penalised Cox regression form, as implemented in the *R* package *GLMNET* [14]. Cox regression fits the model by setting the model coefficients so as to maximise the partial likelihood, as defined by Eq (1),

$$\begin{array}{c} L\left(\theta \right)=\prod_{j\in S}\frac{e^{\theta^{⊤}x_{j}}}{\sum_{j^{\prime }\in R_{j}}e^{\theta^{⊤}x_{j^{\prime }}}}, \end{array}$$ (1)

where ***θ*** denotes the vector of model coefficients, **x**_*j*_ and **x**_*j*′_ are the vectors of predictor variable values for samples *j* and *j*′ respectively (here, per-gene methylation measures), *S* is the set of patients who died during the study, and *R*_*j*_ is the set of samples ‘at risk’ during the time interval when patient *j* died, defined as *R*_*j*_ = {*j*′|*Y*_*j*′_ ≥ *Y*_*j*_}, where *Y*_*j*_ and *Y*_*j*′_ are the times of death of patients *j* and *j*′ respectively. The Elastic Net penalises the log-likelihood corresponding to Eq (1), constraining it according to the magnitude of the model fit coefficients, by subtracting this constraint from the likelihood; in doing so, it ‘chooses’ the best combination of predictor variables (per-gene methylation measures), by adjusting the corresponding model coefficients, and setting these coefficients to zero where the variables provide no useful information or redundant information. The constraint is a combination of some multiples of the *L*_1_ and *L*_2_ norms of the model fit coefficients; the severity and balance of the constraint is controlled by the parameters *λ* (a ‘magnitude’ parameter) and *α* (a ‘blending’ parameter). Hence, the Elastic Net Cox model is fitted by finding model coefficients $\hat{\theta }$ which maximise the penalised log likelihood *ϕ*(***θ***, *λ*, *α*) in Eq (2),

$$\begin{array}{c} \phi \left(\theta ,\lambda ,\alpha \right)=\frac{2}{N}l\left(\theta \right)-\lambda \left({\alpha ∥\theta ∥}_{L_{1}}+\frac{\left(1-\alpha \right)}{2}{∥\theta ∥}_{L_{2}}^{2}\right), \end{array}$$ (2)

where *N* is the number of samples, ∥ ⋅ ∥_*L*_1__ and ∥ ⋅ ∥_*L*_2__ are the *L*_1_ and *L*_2_ norms, and *l*(***θ***) = log(*L*(***θ***)). The *R* package *GLMNET* used for these model fits sets the *λ* parameter internally using ten-fold cross validation, and requires the user to set the *α* parameter (0 ≤ *α* ≤ 1), which was in this case set by choosing the value which minimises the model error after trialling values from 0 to 1 in evenly-spaced intervals of 0.1. Model fitting in this way leads to a set of model coefficients $\hat{\theta }$ for a particular set of predictors (i.e., genome-wide per-gene methylation measures), with one coefficient per predictor, defining those predictors which are present in the model (i.e., predictors with corresponding non-zero coefficients), and their relative weightings.

The fitted model coefficients $\hat{\theta }$ calculated according to Eqs (1) and (2) and the training set data were used to calculate a score ${\hat{\theta }}^{⊤}x_{j}$ for each patient *j*, based on the corresponding per-gene methylation measures **x**_*j*_. These scores were then used to divide the training set into tertiles, defining high and low risk groups. The cutoffs defining the top and bottom tertiles in the training set were then used to divide the test set into three portions, and those most and least at risk (i.e., those test set patients with scores above the top cutoff, and below the bottom cutoff) were compared by Mantzel-Haenszel test, stratified for age, stage and residual disease (disease grade was not associated with survival for this data set), to assess the ability of this model fit to blindly predict patient survival, adjusted for significant clinical covariates. Upper and lower tertiles were compared here as previously by other authors [9] for the OC prognostic signature generation, and the reasoning for doing so in this discovery stage, rather than comparing two groups separated by the median score, was in order to prioritise larger effect sizes. If the samples were split into two groups divided by the median score, relatively small differences in the per-gene methylation measures used to generate this score might result in patients being categorised as high or low risk, with corresponding significant test results from this small variation between patients. Comparison of upper and lower tertiles would be expected to be more robust / stable with respect to such small differences in per-gene methylation measures.

Due to the heterogeneity in the main OC data set which was used to generate the OC prognostic signature, each randomly-selected training set which the Elastic Net model was fitted to lead to a different set of genes being chosen. In order to infer a consistent OC prognostic signature from this data set, i.e., a consensus, the same process of randomly partitioning the data and fitting the model was repeated a total of 10^5^ times for the gene-body IGV measure. Of these, 8281 model fits were able to significantly predict survival in the respective test set (FDR *q* < 0.1). To generate the OC prognostic signature, genes were first ranked by how many of these 8281 significant model fits they appeared in. In the case of ties, genes were additionally ranked by, for each model fit, calculating the proportion of the sum of the absolute coefficient values for that model, which each gene selected as part of that model accounted for, and then comparing, for each tied gene, the mean of these proportions for that gene, across all the models it was selected as part of. Genes were assigned significance according to how many models they were selected as being part of, *y*, out of the total *k* = 8281 models selected as significantly associated with survival, under the null hypothesis that they were present in these observed *y* significant model fits by chance. If there were the same number of genes selected as part of each of these 8281 model fits, then this significance under the null hypothesis might be modelled by a binomial distribution, with the probability *p*_*b*_ of any gene being selected by chance as part of one model fit approximated by *p*_*b*_ = *f*/*m*, where *f* is the number of genes selected as part of each and every model fit, and *m* is the total number of genes for which gene-body methylation variance information is available. The probability of seeing a gene purely by chance in at least *y* model fits, out of a possible total of *k*, with constant probability *p*_*b*_ of appearing in each of these *k* models, would then be given by Eq (3),

$$\begin{array}{c} P\left(Y\geq y\right)=\sum_{r=y}^{k}\left[\left(\begin{array}{l} k\\ r \end{array}\right)p_{b}^{r}{\left(1-p_{b}\right)}^{\left(k-r\right)}\right]. \end{array}$$ (3)

**Fig 7:**
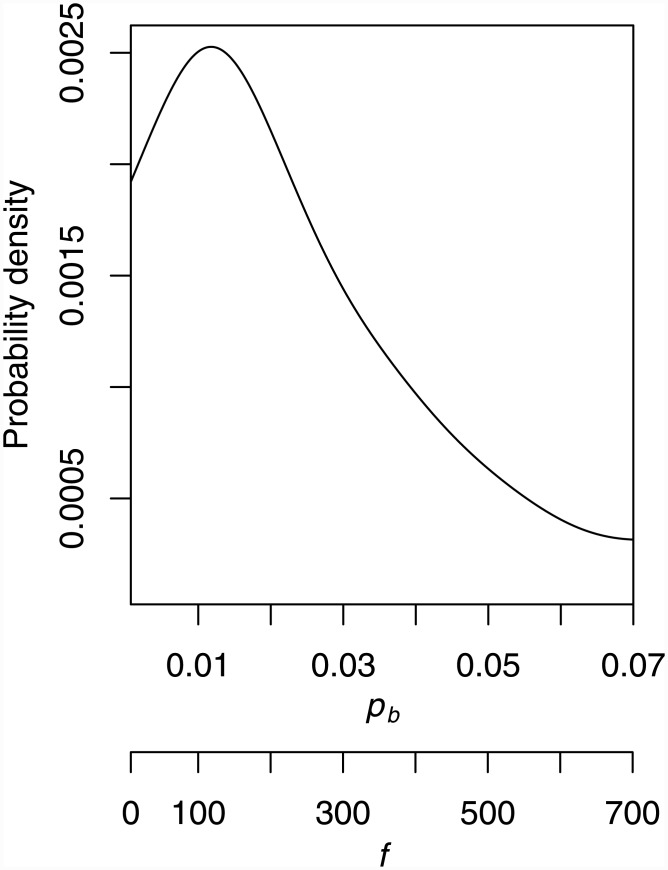
Probability density distribution of the probabilities of a gene being included in a fitted model. The plot shows a kernel-smoothed empirical estimate of the probability density distribution of the number of genes included in each model, *f*, over the 8281 significant gene body methylation variance model fits, with corresponding probability of a gene being included in a model *p*_*b*_ = *f*/*m*, where *m* is the number of genes with gene body methylation variance information available.

However, the number of genes selected, *f*, as part of each model, varies considerably (from 7 to 1697), and consequently *p*_*b*_ cannot be assumed to be constant. Alternatively, *p*_*b*_ could be modelled as being variable and bounded on [0, 1], with a corresponding probability distribution *π*_*b*_ (*p*_*b*_). The distribution *π*_*b*_ (*p*_*b*_) can be estimated as the observed distribution of *f* among the *k* = 8281 significant model fits, again using *p*_*b*_ = *f*/*m*. This leads to a modelled probability, Eq (4), of seeing any gene at least *y* times out of *k* model fits purely by chance, with *p*_*b*_ variable and with its distribution *π*_*b*_(*p*_*b*_) empirically estimated as ${\hat{\pi }}_{b}\left(p_{b}\right)$,

$$\begin{array}{c} P\left(Y\geq y\right)=\sum_{r=y}^{k}\left\{\int_{0}^{1}{\hat{\pi }}_{b}\left(p_{b}\right)\left[\left(\begin{array}{l} k\\ r \end{array}\right)p_{b}^{r}{\left(1-p_{b}\right)}^{\left(k-r\right)}\right]dp_{b}\right\}, \end{array}$$ (4)

with the square brackets included in Eq (4) to highlight the comparison with Eq (3). In practice, the integral in Eq (4) is replaced with a sum over the observed values of *p*_*b*_, as calculated from the observed values of *f*, which range between 7 and 1697. A kernel-smoothed plot of ${\hat{\pi }}_{b}\;\left(p_{b}\right)$, the empirical probability density distribution of *f* and corresponding *p*_*b*_, appears in Fig 7.

### Calculation of the DNAm IGV ovarian cancer prognostic score

Clustering was performed to identify groups of genes in the OC prognostic signature with similar patterns of IGV across patients. The clustering was carried out separately for genes individually associated with worse patient survival outcome for increased IGV (‘hyper’ genes) and for decreased IGV (‘hypo’ genes). Consensus clustering [19] was used for the clustering, with a hierarchical clustering inner loop, using 1 − *ρ* as the distance measure, where *ρ* is the Spearman rank correlation coefficient. The following additional settings were used: probability of selecting a sample = 0.8, probability of selecting a feature = 1, number of resamplings = 10^5^, maximum number of clusters = 20.

The discovered clusters were then filtered (to remove noise, and uncertainty associated with trends inferred from small groups of genes in these genome-wide data), retaining only those clusters which contained at least 10 genes, and only those clusters with mean IGV significantly associated with patient survival outcome. After filtering, four clusters remained, for two of which an increase in the cluster mean IGV was associated with worse patient survival outcome (called ‘hyper 1’ and ‘hyper 2’), and for two of which a decrease in the cluster mean IGV was associated with worse survival outcome (called ‘hypo 1’ and ‘hypo 2’). The IGV cluster scores were then calculated, as the means of the IGV of the genes each of these four clusters.

In order to calculate the IGV prognostic score from these components, a Cox model (adjusted for clinical covariates) was fitted to these four IGV cluster scores. The coefficients for this model (standardised by the variance of the predictors) are fairly similar for each of the clusters (hyper 1: 0.22; hyper 2: 0.25; hypo 1: 0.23; hypo 2: 0.30), indicating that each cluster is important to the model, and to the prognostic predictions. The median of the IGV prognostic score calculated from this Cox model was used to divide the 218 patients in the main DNAm OC data-set used to derive the OC prognostic signature, into better and worse prognostic groups.

### Validation of the ovarian cancer prognostic signature

The DNAm prognostic signature derived from the OC data set was validated in two independent DNAm data sets. The first of these data sets was taken from another study of OC (*n* = 198), and was supplied by the Mayo Clinic. The second of these data sets was taken from a study of uterine corpus endometrioid carcinoma (UCEC) (*n* = 358), and was downloaded from *The Cancer Genome Atlas* (TCGA) project [21].

The IGV prognostic score was similarly calculated by fitting a Cox model to the four IGV cluster scores in the main OC DNAm data set, adjusted for clinical covariates, then applying this model to the equivalent IGV cluster scores in the Mayo Clinic OC and the TCGA UCEC validation sets. In order to make prognostic predictions in these independent data sets using only the DNAm data, the model was used to calculate the IGV prognostic score for the samples in the independent data sets from the fitted model coefficients corresponding to IGV cluster scores only, and not the clinical covariates. This IGV prognostic score was used to define better and worse prognostic groups in the independent data sets, separated by the median IGV prognostic score in the main OC data set. These prognostic groups were then compared, assessing statistical significance with univariate and multivariate Cox regression (i.e., respectively without and with adjustment for the clinical covariates).

### Comparison of IGV with Intra-Tumour Heterogeneity

Intra-tumour methylation heterogeneity was assessed in terms of cross-sample variability of methylation, where the samples are taken from the same patient. The resulting patterns and relationships are compared with cross-sample variability of methylation in the main OC data set, where the samples are now from different patients. Cross-sample variability of methylation is estimated by first calculating the variance of the methylation measurements across all samples for a particular probe, and then taking the mean of these probe variances for all the probes in a gene (gene body probes only). This mean cross-sample methylation variance was compared to the mean IGV of the same gene, which is calculated by taking the mean of the IGV for that gene across the same samples as were used to calculate the cross-sample methylation variance. Cross-sample methylation variance was then analysed as a function of IGV by estimating E(y|x), where *y* represents cross-sample methylation variance and *x* represents IGV, by fitting spline curves. This resulted in one best-fit curve per patient for the EC and prostate cancer intra-tumour heterogeneity datasets, and one best-fit curve for all the patients for each of the main OC and TCGA BRCA basal datasets.

### Testing Transcription-factor binding correlation with IGV

We examined transcription factor binding to the OC prognostic signature genes, using the ENCODE (Encyclopedia of DNA Elements) chromatin immunoprecipitation (ChIP) data [24], with the ANNOVAR software [48]. Transcription factor binding site information was available, for the gene body regions defined, for 55 transcription factors. Each of these TFs was tested for significant over or under enrichment binding to the genes of each of the four prognostic signature clusters, with Fisher’s exact test. We also tested the correlation of the expression level of each of these 55 TFs, with the IGV of genes the TF binds to, and the genes the TF does not bind to. We used a Kolmogorov-Smirnov test to assess whether, for each TF, there is significantly more positive, or more negative, correlation with IGV of the genes it binds to, compared to genes it does not. For this expression correlation analysis, we used the 42 TCGA BRCA basal samples with both expression and DNAm data available, because it was comprehensively demonstrated by the TCGA consortium that high-grade serous ovarian and uterine and BRCA basal cancers are extremely molecularly similar [25], and we also established a high degree of similarity of behaviour between our prognostic signature genes in OC, and these TCGA BRCA basal samples.

### Ethics Statement

The use of tumour tissue has been approved by the local ethical committees of the contributing institutions: Studying the samples contributed from Rotterdam has been approved by the local Rotterdam Medical Ethics Committee (MEC-2008-183), performed in accordance with the Code of Conduct of the Federation of Medical Scientific Societies in the Netherlands (http://www.fmwv.nl). The Regional Committee for Medical Research Ethics in Norway approved the study (for ovarian cancer patients diagnosed in Oslo before 2007, exemption from obtaining informed consent was received as the majority of ovarian cancer patients were dead at the time the application was evaluated; patients diagnosed after 2007 signed general consent allowing for use of the tumours for research purposes). Written informed consent for the use of tumour tissue and prospective clinical data collection was obtained from all patients and approved by the Leuven ethics committee. The use of cancer samples from Bergen was approved by the Regional Research Ethics Committee in Medicine and patients have given their written informed consent to use their sample for research. Patients whose samples were used from the Mayo Clinic gave informed consent and the Mayo Clinic Institutional Review Board approved the study. No identifying patient information was available to us. The data have not been published before.

## Supporting Information

S1 Fig Mean IGV across patients, for the genes of each cluster. (TIF)

S2 Fig Mean gene-body methylation level, across patients, for the genes of each cluster. (PDF)

S1 File Supplementary Tables. (PDF)

## Abbreviations

BRCA: Breast cancer invasive carcinoma
DNAm: DNA methylation
EC: Endometrial cancer
ENCODE: Encyclopedia of DNA elements
FDR: False discovery rate
ITH: Intra-tumour heterogeneity
OC: Ovarian cancer
IGV: Intra gene variability of DNA methylation
TCGA: The Cancer Genome Atlas
TF: Transcription factor
UCEC: Uterine corpus endometrial carcinoma
