# Supplementary figures and images for: Intra-Gene DNA Methylation Variability Is a Clinically Independent Prognostic Marker in Women’s Cancers
Source: PLoS One. 2015 Dec 2;10(12):e0143178. doi: 10.1371/journal.pone.0143178 (PMC4667934; doi:10.1371/journal.pone.0143178)

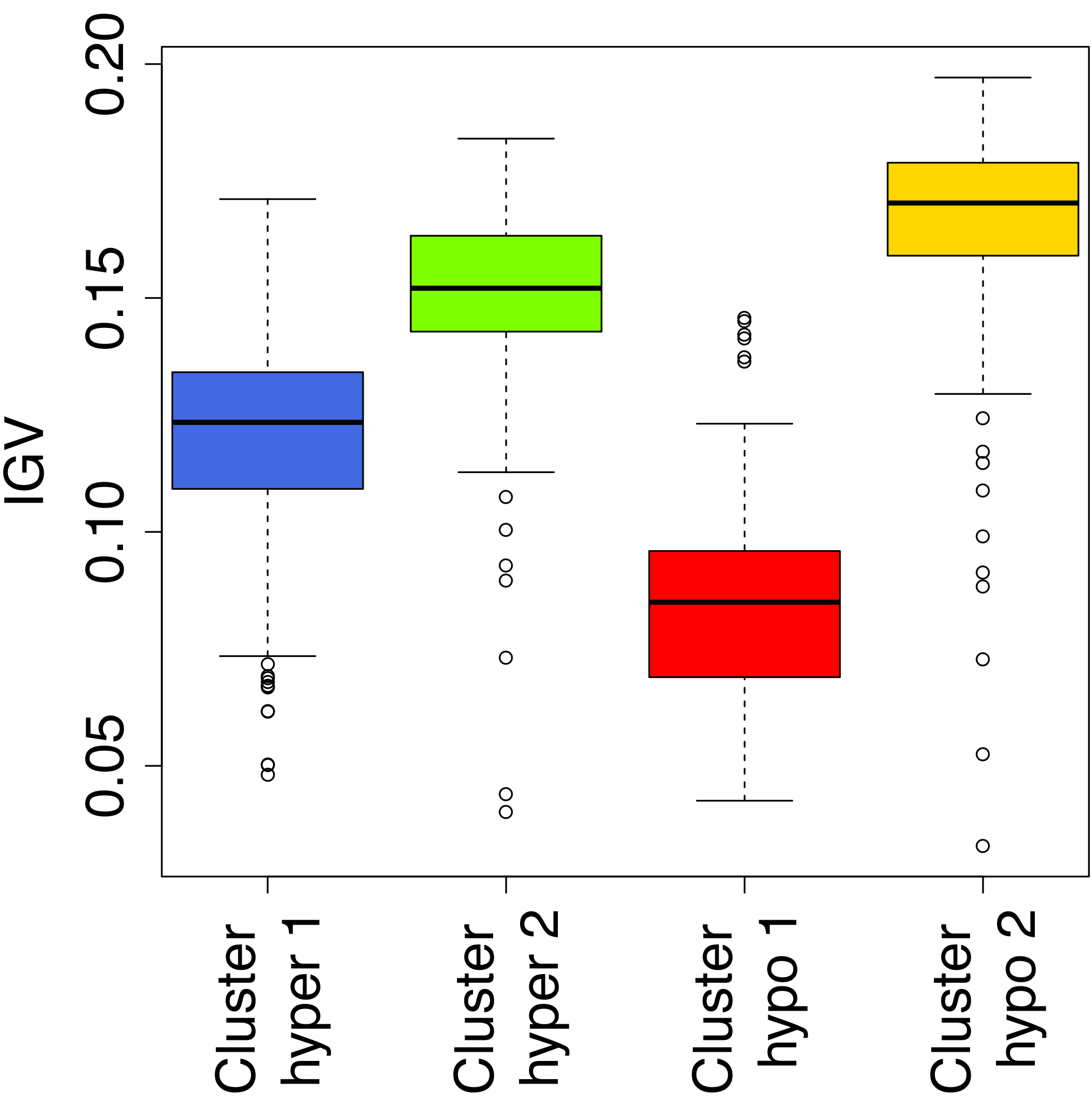

Supplement: S1 Fig — (TIF) [file pone.0143178.s001.tif]

Mean gene body  $\beta$

0.3 0.4 0.5 0.6 0.7

Cluster  
hyper 1

Cluster  
hyper 2

Cluster  
hypo 1

Cluster  
hypo 2

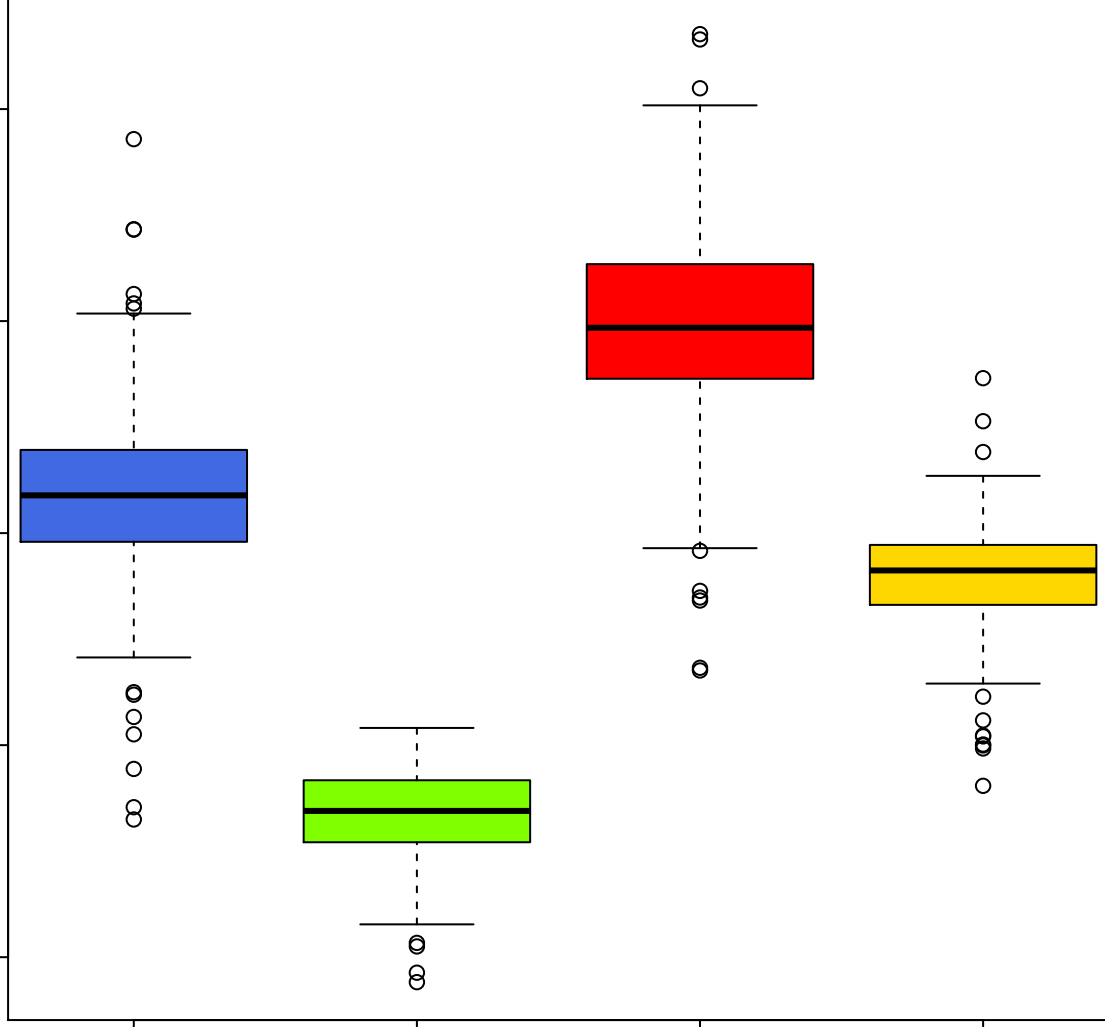

Supplement: S2 Fig — (PDF) [file pone.0143178.s002.pdf]
