# Supplementary material for: Intra-Gene DNA Methylation Variability Is a Clinically Independent Prognostic Marker in Women’s Cancers
Source: PLoS One. 2015 Dec 2;10(12):e0143178. doi: 10.1371/journal.pone.0143178 (PMC4667934; doi:10.1371/journal.pone.0143178)
Supplement: S1 File — (PDF) [file pone.0143178.s003.pdf]

Supplementary Information: Intra-gene DNA methylation variability is a technically and clinically independent prognostic marker in women's cancers

Thomas E. Bartlett, Allison Jones, Ellen L. Goode, Brooke L. Fridley, Julie M Cunningham, Els M. J. J. Berns, Elisabeth Wik, Helga B. Salvesen, Ben Davidson, Claes G. Trope, Sandrina Lambrechts, Ignace Vergote and Martin Widschwendter

Gene	No. models sig, out of 8281	*p*-val	*q*-val	Chr	Gene info
SEMA4A	8274	0	0	1	sema domain, immunoglobulin domain (Ig), transmembrane domain (TM) and short cytoplasmic domain, (semaphorin) 4A
PYCARD	8196	0	0	16	PYD and CARD domain containing
RNF122	8129	0	0	8	ring finger protein 122
ALPL	8127	0	0	1	alkaline phosphatase, liver/bone/kidney
RIC3	8108	0	0	11	resistance to inhibitors of cholinesterase 3 homolog (C. elegans)
CXXC4	8075	0	0	4	CXXC finger protein 4
RSPH9	7945	0	0	6	radial spoke head 9 homolog (Chlamydomonas)
TMEM17	7877	0	0	2	transmembrane protein 17
PCTP	7869	0	0	17	phosphatidylcholine transfer protein
UBAP2L	7837	0	0	1	ubiquitin associated protein 2-like
PLDN	7652	0	0	15	biogenesis of lysosomal organelles complex-1, subunit 6, pallidin
ZNF727	7626	0	0	7	zinc finger protein 727
NRL	7583	0	0	14	neural retina leucine zipper
OBFC1	7488	0	0	10	oligonucleotide/oligosaccharide-binding fold containing 1
SERHL2	7245	0	0	22	serine hydrolase-like 2
FOXR1	7220	0	0	11	forkhead box R1
PROSC	7218	0	0	8	proline synthetase co-transcribed homolog (bacterial)
SLC50A1	7207	0	0	1	solute carrier family 50 (sugar transporter), member 1
PDE1B	7143	0	0	12	phosphodiesterase 1B, calmodulin-dependent
MEX3B	7109	0	0	15	mex-3 homolog B (C. elegans)
NOVA1	7003	0	0	14	neuro-oncological ventral antigen 1
EIF2C4	7002	0	0	1	argonaute RISC catalytic component 4
BK250D10	6945	0	0		
SPATA13	6857	0	0	13	spermatogenesis associated 13
C14orf64	6854	0	0	14	chromosome 14 open reading frame 64
ACRBP	6849	0	0	12	acrosin binding protein
CLRN3	6846	0	0	10	clarin 3
ARL10	6839	0	0	5	ADP-ribosylation factor-like 10
PITHD1	6828	0	0	1	PITH (C-terminal proteasome-interacting domain of thioredoxin-like) domain containing 1
CGN	6761	0	0	1	cingulin
SEC14L4	6746	0	0	22	SEC14-like 4 (S. cerevisiae)
HOXB9	6742	0	0	17	homeobox B9
SEC14L2	6700	0	0	22	SEC14-like 2 (S. cerevisiae)
ANKRD13B	6681	0	0	17	ankyrin repeat domain 13B
NINL	6672	0	0	20	ninein-like
COMMD6	6641	0	0	13	COMM domain containing 6
TBC1D20	6589	0	0	20	TBC1 domain family, member 20
IL17REL	6573	0	0	22	interleukin 17 receptor E-like
ENG	6572	0	0	9	endoglin
GPC5	6557	0	0	13	glypican 5
SAMD10	6483	0	0	20	sterile alpha motif domain containing 10
SRC	6482	0	0	20	v-src sarcoma (Schmidt-Ruppin A-2) viral oncogene homolog (avian)
EGR3	6481	0	0	8	early growth response 3
FAM26F	6436	0	0	6	family with sequence similarity 26, member F
TMEM185B	6338	0	0	2	transmembrane protein 185B
SYBU	6326	0	0	8	syntabulin (syntaxin-interacting)
C14orf126	6316	0	0	14	D-tyrosyl-tRNA deacylase 2 (putative)
WDR65	6307	0	0	1	WD repeat domain 65
RPF2	6286	0	0	6	ribosome production factor 2 homolog (S. cerevisiae)
SNRNP27	6277	0	0	2	small nuclear ribonucleoprotein 27kDa (U4/U6.U5)
ATG4D	6213	0	0	19	autophagy related 4D, cysteine peptidase
ABCB6	6158	0	0	2	ATP-binding cassette, sub-family B (MDR/TAP), member 6
IFNGR1	6133	0	0	6	interferon gamma receptor 1
SLC27A6	6132	0	0	5	solute carrier family 27 (fatty acid transporter), member 6
TLE4	6111	0	0	9	transducin-like enhancer of split 4 (E(sp1) homolog, Drosophila)
TCL6	6078	0	0	14	T-cell leukemia/lymphoma 6 (non-protein coding)
ECEL1P2	6047	0	0	2	endothelin converting enzyme-like 1, pseudogene 2
LOC100134259	6006	0	0	2	uncharacterized LOC100134259
ZNF300P1	5956	0	0	5	zinc finger protein 300 pseudogene 1
SP5	5944	0	0	2	Sp5 transcription factor
ICOSLG	5928	0	0	21	inducible T-cell co-stimulator ligand
CENPW	5699	0	0	6	centromere protein W
GAMT	5692	0	0	19	guanidinoacetate N-methyltransferase
IQCG	5655	0	0	3	IQ motif containing G
PIK3R3	5641	0	0	1	phosphoinositide-3-kinase, regulatory subunit 3 (gamma)
EGR2	5631	0	0	10	early growth response 2
GRASP	5628	0	0	12	GRP1 (general receptor for phosphoinositides 1)-associated scaffold protein
LOC391322	5608	0	0	22	D-dopachrome tautomerase-like
HRASLS5	5577	0	0	11	HRAS-like suppressor family, member 5
GPNMB	5576	0	0	7	glycoprotein (transmembrane) nmb
B4GALNT1	5542	0	0	12	beta-1,4-N-acetyl-galactosaminyl transferase 1
TCFL5	5518	0	0	20	transcription factor-like 5 (basic helix-loop-helix)
C1orf173	5501	0	0	1	chromosome 1 open reading frame 173
NOX4	5468	0	0	11	NADPH oxidase 4
ZBTB10	5461	0	0	8	zinc finger and BTB domain containing 10
VSTM5	5431	0	0	11	V-set and transmembrane domain containing 5
RPS7	5403	0	0	2	ribosomal protein S7
COL17A1	5360	0	0	10	collagen, type XVII, alpha 1
ZNF346	5324	0	0	5	zinc finger protein 346
CHSY3	5308	0	0	5	chondroitin sulfate synthase 3
ARMC7	5287	0	0	17	armadillo repeat containing 7
ARSA	5238	0	0	22	arylsulfatase A
PLA2G16	5232	0	0	11	phospholipase A2, group XVI
CD74	5226	0	0	5	CD74 molecule, major histocompatibility complex, class II invariant chain
GCM2	5202	0	0	6	glial cells missing homolog 2 (Drosophila)
HNRNPA3	5201	0	0	2	heterogeneous nuclear ribonucleoprotein A3
LGALS9	5154	0	0	17	lectin, galactoside-binding, soluble, 9
KCNJ9	5135	0	0	1	potassium inwardly-rectifying channel, subfamily J, member 9
MYOM1	5095	0	0	18	myomesin 1
NAA40	5082	0	0	11	N(alpha)-acetyltransferase 40, NatD catalytic subunit, homolog (S. cerevisiae)
MS4A15	5073	0	0	11	membrane-spanning 4-domains, subfamily A, member 15
SERHL	5069	0	0	22	serine hydrolase-like
SYT14	5036	0	0	1	synaptotagmin XIV
MYO3A	5005	0	0	10	myosin IIIA
SLC22A20	4997	0	0	11	solute carrier family 22, member 20
XRCC2	4953	0	0	7	X-ray repair complementing defective repair in Chinese hamster cells 2
PLK2	4942	0	0	5	polo-like kinase 2
TCP11	4902	0	0	6	t-complex 11, testis-specific
TFPI2	4885	0	0	7	tissue factor pathway inhibitor 2
GOLPH3	4864	0	0	5	golgi phosphoprotein 3 (coat-protein)

Supplementary Table 1: Ovarian cancer prognostic signature - top 100 genes.

Significance is assigned to genes according to the frequency with which they appear in model fits which are significantly predictive of patient outcome (survival, adjusted for clinical covariates). Gene body DNA methylation variance was used as a per-gene measure for the model fits.

	Symbol	Rank in prog. sig.	q-val	Chr	Info
1	*SEMA4A*	1	0	1	sema domain, immunoglobulin domain (Ig), transmembrane domain (TM) and short cytoplasmic domain, (semaphorin) 4A
2	*RSPH9*	7	0	6	radial spoke head 9 homolog (Chlamydomonas)
3	*C14orf64*	25	0	14	chromosome 14 open reading frame 64
4	*SRC*	42	0	20	v-src sarcoma (Schmidt-Ruppin A-2) viral oncogene homolog (avian)
5	*C1orf173*	73	0	1	chromosome 1 open reading frame 173
6	*CHSY3*	80	0	5	chondroitin sulfate synthase 3
7	*MYO3A*	94	0	10	myosin IIIA
8	*PLK2*	97	0	5	polo-like kinase 2
9	*GPR137B*	127	0	1	G protein-coupled receptor 137B
10	*ABCA8*	137	0	17	ATP-binding cassette, sub-family A (ABC1), member 8
11	*HERC5*	141	0	4	HECT and RLD domain containing E3 ubiquitin protein ligase 5
12	*TMEM101*	154	0	17	transmembrane protein 101
13	*FAM162B*	158	0	6	family with sequence similarity 162, member B
14	*KANK1*	161	0	9	KN motif and ankyrin repeat domains 1
15	*TUBB2B*	176	0	6	tubulin, beta 2B class IIb
16	*PLK1*	191	0	16	polo-like kinase 1
17	*LHCGR*	194	0	2	luteinizing hormone/choriogonadotropin receptor
18	*C1QL3*	225	0	10	complement component 1, q subcomponent-like 3
19	*RHPN2*	240	0	19	rhophilin, Rho GTPase binding protein 2
20	*PARP15*	298	1.1e-166	3	poly (ADP-ribose) polymerase family, member 15
21	*IRF4*	307	3.9e-152	6	interferon regulatory factor 4
22	*COL6A5*	326	7.1e-124	3	collagen, type VI, alpha 5
23	*KBTBD8*	335	1.4e-115	3	kelch repeat and BTB (POZ) domain containing 8
24	*MLF1*	345	6.8e-103	3	myeloid leukemia factor 1
25	*PTH2R*	359	3.6e-81	2	parathyroid hormone 2 receptor
26	*ACHE*	364	2e-74	7	acetylcholinesterase
27	*C6orf97*	379	1.3e-58	6	coiled-coil domain containing 170
28	*GRB14*	390	1.3e-47	2	growth factor receptor-bound protein 14
29	*NPR3*	416	8.2e-34	5	natriuretic peptide receptor C/guanylate cyclase C (atrionatriuretic peptide receptor C)
30	*C20orf194*	438	2.5e-24	20	chromosome 20 open reading frame 194
31	*NID2*	446	2.4e-21	14	nidogen 2 (osteonidogen)
32	*KCNMB4*	471	5.1e-11	12	potassium large conductance calcium-activated channel, subfamily M, beta member 4
33	*B3GNT9*	476	1.7e-10	16	UDP-GlcNAc:betaGal beta-1,3-N-acetylglucosaminyltransferase 9
34	*NPTX1*	483	3.1e-09	17	neuronal pentraxin I
35	*SHISA9*	499	1.6e-06	16	shisa homolog 9 (Xenopus laevis)
36	*ASIP*	509	1.7e-05	20	agouti signaling protein
37	*CCND2*	519	0.00011	12	cyclin D2
38	*SNX18*	524	0.00021	5	sorting nexin 18
39	*CPA2*	529	4e-04	7	carboxypeptidase A2 (pancreatic)
40	*PRR25*	533	0.00047	16	proline rich 25
41	*DUSP27*	535	5e-04	1	dual specificity phosphatase 27 (putative)
42	*CD302*	546	0.00087	2	CD302 molecule
43	*SLC12A8*	549	0.001	3	solute carrier family 12 (potassium/chloride transporters), member 8
44	*PHACTR3*	553	0.0011	20	phosphatase and actin regulator 3
45	*OTX2*	569	0.0026	14	orthodenticle homeobox 2
46	*CAV2*	573	0.0035	7	caveolin 2
47	*PALM3*	577	0.004	19	paralemmin 3
48	*SEZ6L2*	578	0.0042	16	seizure related 6 homolog (mouse)-like 2
49	*SUN3*	601	0.01	7	Sad1 and UNC84 domain containing 3
50	*EPHA6*	612	0.013	3	EPH receptor A6
51	*HLA-F-AS1*	628	0.018	6	HLA-F antisense RNA 1
52	*ALDH1A2*	629	0.019	15	aldehyde dehydrogenase 1 family, member A2
53	*TRPC1*	637	0.022	3	transient receptor potential cation channel, subfamily C, member 1
54	*TSHZ3*	646	0.029	19	teashirt zinc finger homeobox 3
55	*ITGA4*	648	0.031	2	integrin, alpha 4 (antigen CD49D, alpha 4 subunit of VLA-4 receptor)
56	*NEGR1*	653	0.033	1	neuronal growth regulator 1

Supplementary Table 2: Prognostic signature, cluster ‘hyper 1’

	Symbol	Rank in prog. sig.	q-val	Chr	Info
1	*RNF122*	3	0	8	ring finger protein 122
2	*PLDN*	11	0	15	biogenesis of lysosomal organelles complex-1, subunit 6, pallidin
3	*EIF2C4*	22	0	1	argonaute RISC catalytic component 4
4	*TBC1D20*	37	0	20	TBC1 domain family, member 20
5	*C14orf126*	47	0	14	D-tyrosyl-tRNA deacylase 2 (putative)
6	*RPF2*	49	0	6	ribosome production factor 2 homolog (S. cerevisiae)
7	*SNRNP27*	50	0	2	small nuclear ribonucleoprotein 27kDa (U4/U6.U5)
8	*TLE4*	55	0	9	transducin-like enhancer of split 4 (E(sp1) homolog, Drosophila)
9	*CENPW*	62	0	6	centromere protein W
10	*PIK3R3*	65	0	1	phosphoinositide-3-kinase, regulatory subunit 3 (gamma)
11	*RPS7*	77	0	2	ribosomal protein S7
12	*PLA2G16*	83	0	11	phospholipase A2, group XVI
13	*HNRNPA3*	86	0	2	heterogeneous nuclear ribonucleoprotein A3
14	*XRCC2*	96	0	7	X-ray repair complementing defective repair in Chinese hamster cells 2
15	*GOLPH3*	100	0	5	golgi phosphoprotein 3 (coat-protein)
16	*CHORDC1*	110	0	11	cysteine and histidine-rich domain (CHORD) containing 1
17	*GFPT1*	120	0	2	glutamine–fructose-6-phosphate transaminase 1
18	*CISD1*	129	0	10	CDGSH iron sulfur domain 1
19	*TRA2A*	133	0	7	transformer 2 alpha homolog (Drosophila)
20	*MKRN2*	134	0	3	makorin ring finger protein 2
21	*CAD*	144	0	2	carbamoyl-phosphate synthetase 2, aspartate transcarbamylase, and dihydroorotase
22	*SPG11*	157	0	15	spastic paraplegia 11 (autosomal recessive)
23	*RNF34*	177	0	12	ring finger protein 34, E3 ubiquitin protein ligase
24	*SERINC3*	188	0	20	serine incorporator 3
25	*C3orf38*	192	0	3	chromosome 3 open reading frame 38
26	*MYC*	203	0	8	v-myc myelocytomatosis viral oncogene homolog (avian)
27	*RPE*	204	0	2	ribulose-5-phosphate-3-epimerase
28	*SNRPG*	210	0	2	small nuclear ribonucleoprotein polypeptide G
29	*HSF2*	216	0	6	heat shock transcription factor 2
30	*DSCR3*	219	0	21	Down syndrome critical region gene 3
31	*DEPTOR*	226	0	8	DEP domain containing MTOR-interacting protein
32	*SLC30A6*	238	0	2	solute carrier family 30 (zinc transporter), member 6
33	*SRP19*	239	0	5	signal recognition particle 19kDa
34	*YEATS4*	252	3.4e-313	12	YEATS domain containing 4
35	*PYGL*	259	4.6e-296	14	phosphorylase, glycogen, liver
36	*POLR3H*	264	1.6e-280	22	polymerase (RNA) III (DNA directed) polypeptide H (22.9kD)
37	*FAM65B*	274	2.1e-220	6	family with sequence similarity 65, member B
38	*PVALB*	277	5.5e-217	22	parvalbumin
39	*FAM98A*	293	1.5e-180	2	family with sequence similarity 98, member A
40	*RAB8A*	294	1.7e-176	19	RAB8A, member RAS oncogene family
41	*TTC17*	301	2.3e-163	11	tetratricopeptide repeat domain 17
42	*CWC25*	308	4.1e-150	17	CWC25 spliceosome-associated protein homolog (S. cerevisiae)
43	*EIF4EBP2*	309	4.2e-146	10	eukaryotic translation initiation factor 4E binding protein 2
44	*TXNL1*	311	4.2e-146	18	thioredoxin-like 1
45	*SPAG9*	319	3.6e-132	17	sperm associated antigen 9
46	*STXBP3*	324	3.5e-125	1	syntaxin binding protein 3
47	*CLIC4*	331	1.1e-119	1	chloride intracellular channel 4
48	*PSMC6*	334	4.3e-117	14	proteasome (prosome, macropain) 26S subunit, ATPase, 6
49	*ITM2B*	336	2.7e-114	13	integral membrane protein 2B
50	*SFT2D2*	343	2.3e-104	1	SFT2 domain containing 2
51	*CWF19L1*	348	2.6e-95	10	CWF19-like 1, cell cycle control (S. pombe)
52	*HMGN1*	350	3.2e-93	21	high mobility group nucleosome binding domain 1
53	*CYB5R1*	351	1.6e-92	1	cytochrome b5 reductase 1
54	*LOC153684*	355	1.6e-87	5	uncharacterized LOC153684
55	*NKX2-2*	361	1.2e-75	20	NK2 homeobox 2
56	*NDUFA11*	363	1.3e-74	19	NADH dehydrogenase (ubiquinone) 1 alpha subcomplex, 11, 14.7kDa
57	*CDKN2C*	366	3.7e-72	1	cyclin-dependent kinase inhibitor 2C (p18, inhibits CDK4)
58	*LOC387647*	370	9.4e-67	10	patched domain containing 3 pseudogene 1
59	*MSH6*	372	1.3e-64	2	mutS homolog 6 (E. coli)
60	*NEMF*	388	9.1e-48	14	nuclear export mediator factor
61	*PAPOLA*	398	1.1e-44	14	poly(A) polymerase alpha
62	*C8orf38*	399	2.3e-44	8	NADH dehydrogenase (ubiquinone) complex I, assembly factor 6
63	*CCDC138*	406	1.2e-40	2	coiled-coil domain containing 138
64	*KIAA1429*	407	1.8e-40	8	KIAA1429
65	*HDAC2*	408	2e-39	6	histone deacetylase 2
66	*TNFSF9*	411	5.7e-39	19	tumor necrosis factor (ligand) superfamily, member 9
67	*CLTA*	421	1.9e-32	9	clathrin, light chain A
68	*MAPK12*	424	1.2e-31	22	mitogen-activated protein kinase 12
69	*SCFD1*	428	4.8e-29	14	sec1 family domain containing 1
70	*PARP11*	440	7.1e-24	12	poly (ADP-ribose) polymerase family, member 11
71	*UGGT1*	442	5.7e-23	2	UDP-glucose glycoprotein glucosyltransferase 1
72	*MICALCL*	443	1.5e-22	11	MICAL C-terminal like
73	*MPHOSPH8*	457	1.5e-15	13	M-phase phosphoprotein 8
74	*SUZ12P*	466	1.1e-13	17	suppressor of zeste 12 homolog pseudogene 1
75	*EPHX2*	468	2.2e-13	8	epoxide hydrolase 2, cytoplasmic
76	*MAT2B*	490	1.1e-08	5	methionine adenosyltransferase II, beta
77	*C6orf223*	491	2.7e-08	6	chromosome 6 open reading frame 223
78	*RARS*	492	2e-07	5	arginyl-tRNA synthetase
79	*BCAP29*	496	6.4e-07	7	B-cell receptor-associated protein 29
80	*BBS5*	502	3.8e-06	2	Bardet-Biedl syndrome 5
81	*DONSON*	510	2.1e-05	21	downstream neighbor of SON
82	*TEX14*	543	0.00083	17	testis expressed 14
83	*FAM21C*	547	0.00097	10	family with sequence similarity 21, member C
84	*L3MBTL2*	560	0.0014	22	l(3)mbt-like 2 (Drosophila)
85	*CLCN3*	562	0.0017	4	chloride channel, voltage-sensitive 3
86	*HFE*	575	0.004	6	hemochromatosis
87	*SRSF1*	586	0.0072	17	serine/arginine-rich splicing factor 1
88	*CCNB1*	587	0.0073	5	cyclin B1
89	*SLC30A1*	588	0.0074	1	solute carrier family 30 (zinc transporter), member 1
90	*POLR3B*	591	0.0085	12	polymerase (RNA) III (DNA directed) polypeptide B
91	*C9orf40*	594	0.0089	9	chromosome 9 open reading frame 40
92	*NFU1*	595	0.0093	2	NFU1 iron-sulfur cluster scaffold homolog (S. cerevisiae)
93	*KPNB1*	597	0.0094	17	karyopherin (importin) beta 1
94	*BLOC1S1*	607	0.012	12	biogenesis of lysosomal organelles complex-1, subunit 1
95	*LOC100132215*	614	0.014	2	uncharacterized LOC100132215

Supplementary Table 3: Prognostic signature, cluster ‘hyper 2’ (top 95 genes)

	Symbol	Rank in prog. sig.	q-val	Chr	Info
1	*ZNF727*	12	0	7	zinc finger protein 727
2	*PDE1B*	19	0	12	phosphodiesterase 1B, calmodulin-dependent
3	*BK250D10*	23	0		
4	*ANKRD13B*	34	0	17	ankyrin repeat domain 13B
5	*IL17REL*	38	0	22	interleukin 17 receptor E-like
6	*GPC5*	40	0	13	glypican 5
7	*ZNF300P1*	59	0	5	zinc finger protein 300 pseudogene 1
8	*EGR2*	66	0	10	early growth response 2
9	*GPNMB*	70	0	7	glycoprotein (transmembrane) nmb
10	*B4GALNT1*	71	0	12	beta-1,4-N-acetyl-galactosaminyl transferase 1
11	*KCNJ9*	88	0	1	potassium inwardly-rectifying channel, subfamily J, member 9
12	*LTF*	102	0	3	lactotransferrin
13	*FSCN2*	105	0	17	fascin homolog 2, actin-bundling protein, retinal (Strongylocentrotus purpuratus)
14	*GPRIN1*	112	0	5	G protein regulated inducer of neurite outgrowth 1
15	*ZDHHC22*	116	0	14	zinc finger, DHHC-type containing 22
16	*RGR*	128	0	10	retinal G protein coupled receptor
17	*UFD1L*	130	0	22	ubiquitin fusion degradation 1 like (yeast)
18	*AQP2*	147	0	12	aquaporin 2 (collecting duct)
19	*LPAR5*	156	0	12	lysophosphatidic acid receptor 5
20	*ECEL1*	163	0	2	endothelin converting enzyme-like 1
21	*CSF1R*	202	0	5	colony stimulating factor 1 receptor
22	*RALGAPA2*	211	0	20	Ral GTPase activating protein, alpha subunit 2 (catalytic)
23	*KHDRBS2*	245	0	6	KH domain containing, RNA binding, signal transduction associated 2
24	*TMEM26*	258	2.2e-304	10	transmembrane protein 26
25	*RQCD1*	263	4.3e-287	2	RCD1 required for cell differentiation1 homolog (S. pombe)
26	*ADAMTSL3*	269	5.3e-239	15	ADAMTS-like 3
27	*KCNS1*	290	1.7e-188	20	potassium voltage-gated channel, delayed-rectifier, subfamily S, member 1
28	*HOXA9*	297	1.4e-167	7	homeobox A9
29	*LECT1*	317	4.7e-139	13	leukocyte cell derived chemotaxin 1
30	*EPHX3*	330	5.5e-121	19	epoxide hydrolase 3
31	*C12orf56*	360	1.5e-77	12	chromosome 12 open reading frame 56
32	*CDO1*	368	2.5e-69	5	cysteine dioxygenase, type I
33	*LOC644172*	382	8.9e-57	17	mitogen-activated protein kinase 8 interacting protein 1 pseudogene
34	*FAM198B*	397	1.1e-44	4	family with sequence similarity 198, member B
35	*UBE2QL1*	427	8.3e-30	5	ubiquitin-conjugating enzyme E2Q family-like 1
36	*NPY*	441	1.5e-23	7	neuropeptide Y
37	*KLB*	503	5e-06	4	klotho beta
38	*PIF1*	516	8e-05	15	PIF1 5'-to-3' DNA helicase homolog (S. cerevisiae)
39	*SLC10A4*	527	0.00038	4	solute carrier family 10 (sodium/bile acid cotransporter family), member 4
40	*ANXA6*	558	0.0013	5	annexin A6
41	*LINC00271*	565	0.0022	6	long intergenic non-protein coding RNA 271
42	*TSPAN32*	625	0.017	11	tetraspanin 32
43	*TRPM8*	647	0.031	2	transient receptor potential cation channel, subfamily M, member 8
44	*SPHKAP*	656	0.034	2	SPHK1 interactor, AKAP domain containing
45	*RCAN2*	676	0.048	6	regulator of calcineurin 2

Supplementary Table 4: Prognostic signature, cluster 'hypo 1'

	Symbol	Rank in prog. sig.	q-val	Chr	Info
1	*NOX4*	74	0	11	NADPH oxidase 4
2	*CD74*	84	0	5	CD74 molecule, major histocompatibility complex, class II invariant chain
3	*DYRK1A*	167	0	21	dual-specificity tyrosine-(Y)-phosphorylation regulated kinase 1A
4	*CPE*	180	0	4	carboxypeptidase E
5	*MT1F*	267	1.7e-241	16	metallothionein 1F
6	*SPEF2*	276	4.9e-219	5	sperm flagellar 2
7	*NFKBIZ*	287	1e-191	3	nuclear factor of kappa light polypeptide gene enhancer in B-cells inhibitor, zeta
8	*CDR2*	295	3.1e-171	16	cerebellar degeneration-related protein 2, 62kDa
9	*GINS1*	302	3.8e-162	20	GINS complex subunit 1 (Psf1 homolog)
10	*COPZ1*	316	9.9e-141	12	coatomer protein complex, subunit zeta 1
11	*CCDC110*	338	5e-112	4	coiled-coil domain containing 110
12	*NEK9*	393	1.2e-45	14	NIMA-related kinase 9
13	*TMEM170B*	417	1.1e-33	6	transmembrane protein 170B
14	*NRIP3*	448	8e-21	11	nuclear receptor interacting protein 3
15	*NAAA*	464	3.4e-14	4	N-acylethanolamine acid amidase
16	*PPAT*	501	2e-06	4	phosphoribosyl pyrophosphate amidotransferase
17	*VWDE*	579	0.005	7	von Willebrand factor D and EGF domains
18	*SLC16A11*	642	0.027	17	solute carrier family 16, member 11 (monocarboxylic acid transporter 11)
19	*JHDM1D*	652	0.033	7	jumonji C domain containing histone demethylase 1 homolog D (S. cerevisiae)

Supplementary Table 5: Prognostic signature, cluster 'hypo 2'

CGI region	OC prog signat.	Gene body mean	Cluster Hyper1	Cluster Hyper2	Cluster Hyper3	Cluster Hyper4
Island	-2.93% ($p < 0.001$)	3.68% ($p < 0.001$)	1.52% ($p = 0.48$)	7.06% ($p < 0.001$)	7.07% ($p = 0.03$)	-2.08% ($p = 0.628$)
Shore	-1.09% ($p < 0.001$)	0.915% ($p = 0.166$)	-4.62% ($p = 0.016$)	7.47% ($p < 0.001$)	1.18% ($p = 0.614$)	0.0419% ($p = 0.834$)
Shelf	1.26% ($p < 0.001$)	1.19% ($p = 0.01$)	2.95% ($p = 0.778$)	5.07% ($p < 0.001$)	2.84% ($p = 0.09$)	4.2% ($p = 0.15$)

Supplementary Table 6: CpG island enrichment of prognostic signature CpGs.

Relative CpG island enrichment of CpGs mapping to gene bodies was assessed for all genes genome-wide, for the genes of the prognostic signature, and for the genes of the four prognostic clusters. Permutation-test p-values were obtained by randomly sampling the same number of probes annotated to the gene bodies of the genes under consideration, from all probes genome-wide, and repeating this 1000 times.

OC prog signat.	Gene body mean	Cluster Hyper1	Cluster Hyper2	Cluster Hyper3	Cluster Hyper4
4.61% ($p < 0.001$)	6.11% ($p < 0.001$)	-1.14% ($p = 0.57$)	-4.75% ($p = 0.01$)	15.8% ($p < 0.001$)	4.34% ($p = 0.234$)

Supplementary Table 7: Enhancer region enrichment of prognostic signature CpGs.

Relative enhancer-region enrichment of CpGs mapping to gene bodies was assessed for all genes genome-wide, for the genes of the prognostic signature, and for the genes of the four prognostic clusters. Permutation-test p-values were obtained by randomly sampling the same number of probes annotated to the gene bodies of the genes under consideration, from all probes genome-wide, and repeating this 1000 times.
